# Safety and efficacy of ablation for atrial fibrillation in combination with left atrial appendage occlusion in octogenarians

**DOI:** 10.1002/clc.24099

**Published:** 2023-07-31

**Authors:** Peng‐Pai Zhang, Yan Zhao, Jian Sun, Qun‐Shan Wang, Wei Li, Rui Zhang, Mu Chen, Bin‐Feng Mo, Yi Yu, Xiang‐Fei Feng, Bo Liu, Yi‐Chi Yu, Qiu‐Fen Lu, Yi‐Gang Li

**Affiliations:** ^1^ Department of Cardiology, Xinhua Hospital, Shanghai Jiao Tong University School of Medicine Shanghai China

**Keywords:** atrial fibrillation, catheter ablation, left atrial appendage occlusion, octogenarian, stroke

## Abstract

**Background:**

Catheter ablation (CA) combined with left atrial appendage occlusion (LAAO) is a feasible approach for atrial fibrillation (AF) patients. Its role in octogenarians with AF is unclear.

**Hypothesis:**

In AF patients over 80 years, CA combined with LAAO is a feasible way in restoring sinus rhythm and preventing stroke.

**Methods:**

This is a single‐center retrospective study. Patients who underwent CA and LAAO in a single procedure between March 2018 and December 2020 were included. Efficacy endpoints included procedural success rate, AF recurrence rate, and thromboembolic events. Safety endpoints included pericardial effusion/cardiac tamponade, device‐related thrombus (DRT), all‐cause death, and major bleeding.

**Results:**

Five hundred and five patients (mean age 69.5 ± 7.7 years; 230 [45.5%] female) were included, with 46 (9.1%) patients aged ≥80 years old (octogenarian group). Prevalence of paroxysmal AF (25 [54.3%] vs. 207 [45.1%], *p* < 0.001) and CHA2DS2VASc score (4.1 ± 1.3 vs. 3.1 ± 1.4, *p* < 0.0001) were higher in octogenarian patients. There were six cases (1.2%) of pericardial effusion (all in nonoctogenarian patients). At 3 months postprocedure, 437 patients underwent TEE/CT. Thirty‐two (80%) octogenarian patients and 308 (77.6%) nonoctogenarian patients had no peri‐device leak. After a mean follow‐up of 26.9 ± 9.1 months, AF was documented in 10 (21.7%) patients in octogenarian group and in 103 (22.4%) patients in nonoctogenarian group (*p* = 0.99). The annual thromboembolic risk was 2.1% and 0.8% in the octogenarian group and nonoctogenarian group, respectively. Death occurred in 16 nonoctogenarian patients. One major bleeding was recorded in the octogenarian group.

**Conclusions:**

The combination of CA and LAAO in a single procedure is a feasible treatment option in octogenarians with comparable efficacy and safety profile.

## Graphical abstract

Patients aged ≥80 years can undergo catheter ablation in combined with left atrial appendage occlusion in a single procedure safely. This approach can efficiently restore sinus rhythm and reduce stroke risk in elderly patients.

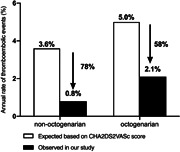

## 1 INTRODUCTION

Atrial fibrillation (AF) is the most common sustained cardiac arrhythmia. The AF lifetime risk is 37.4% at the index age of 55 years and increases with age.^1^, ^2^, ^3^ Patients with AF have a five‐fold higher risk of stroke, increasing up to 16% in high‐risk groups.^4^ Catheter ablation (CA) is an effective treatment for symptomatic AF in restoring sinus rhythm and improving clinical outcomes, but its role in stroke prevention is unproved.^3^, ^5^ Left atrial appendage occlusion (LAAO), in contrast, has demonstrated efficacy for stroke prevention in nonvalvular AF.^6^, ^7^, ^8^, ^9^

Combining CA and LAAO into a single procedure could be a feasible approach in relieving symptoms and reducing the incidence of stroke in high‐risk patients.^10^, ^11^, ^12^, ^13^, ^14^, ^15^, ^16^ Most previous studies analyzed the safety and efficacy based on results derived from small sample size and young patients. AF patients aged ≥80 years (octogenarians) usually have more comorbidities and poor outcome.^17^, ^18^ Significant concerns persist amongst general practitioners for this patient group and they are usually excluded from the combined CA and LAAO procedure.

Clinical data regarding the efficacy and safety of the combined CA and LAAO procedure in this high‐risk group are still far too scanty. In this study, we compared the efficacy and safety of the combined procedure between AF patients aged ≥80 years (octogenarians) and those aged <80 years (nonoctogenarians).

## 2 METHODS

### 2.1 Study design and participants

We retrospectively enrolled all patients who underwent both CA and LAAO in a single procedure between March 2018 and December 2020 in Xinhua Hospital, School of Medicine, Shanghai Jiao Tong University, Shanghai, China. Written informed consent was obtained from all participants. The study was approved by the hospital's ethics committee. Patients' demographics and medical history were collected using patients' chart reviews. Stroke risk according to the CHA2DS2‐VASc and the HAS‐BLED scores was calculated.

All patients discontinued antiarrhythmic drugs (AAD) (except for beta‐blockers) and anti‐thrombotic medications and were bridged with low‐molecular‐weight heparin before the procedure. Transesophageal echocardiography (TEE) and cardiac computed tomography (CCT) were performed to assess the cardiac morphology and exclude LAA thrombus within 48 h before the procedure.

### 2.2 CA for AF

All procedures were performed in patients under conscious sedation. A decapolar catheter was introduced into the coronary sinus and two transseptal accesses were obtained through the right femoral vein. A single heparin bolus of 100 IU/kg was administered following transseptal puncture. During the procedure, the activated clotting time (ACT) was maintained from 250 to 300 s. The CARTO (Biosense Webster) or EnSite (St. Jude Medical) 3‐dimensional electroanatomic mapping systems were used for mapping cardiac reconstruction and guiding ablation. All patients underwent standard pulmonary vein isolation (PVI) and additional liner ablation was performed according to the physician's discretion. The endpoint of ablation was left and right‐sided pulmonary vein electrical isolation and restoring sinus rhythm by either ablation or electric cardioversion. The CA procedure time was determined from the femoral venous puncture to the end of the ablation.

### 2.3 LAAO

Watchman 2.5 device (Boston Scientific) (WM) implantation was performed immediately after the ablation. The initial transseptal sheath was replaced by a 14F access sheath and a pigtail catheter was positioned in the LAA. Angiography was performed to determine the ostial dimension and depth of the LAA. There were 5 device sizes (21, 24, 27, 30, and 33 mm) to accommodate various LAA anatomies and sizes. A device size of 10%–20% greater than the largest diameter of the LAA ostium was chosen. The device was released only if the position‐anchor‐size‐seal (PASS) principle was fulfilled.^15^, ^19^ The compression for Watchman 2.5 device is 8%–20%. Angiography and/or TEE were performed to confirm the appropriate implantation of the device. The LAAO procedure time was determined from the exchange of the WM access sheath to the end of the procedure.

### 2.4 Postprocedure assessment and follow‐up

All patients were anticoagulated (either warfarin [target INR 2.0–3.0] or nonvitamin K antagonist oral anticoagulant [NOAC] at the physician's discretion) for at least 3 months after the procedure. Outpatient visits were scheduled at 3 and 6 months after discharge, every 6 months thereafter, and whenever procedural‐related symptoms arises. A 12‐lead ECG and 24 h Holter monitor were obtained for detecting arrhythmia at each follow‐up. TEE or CCT ^20^, ^21^ was performed at 3 months to evaluate LAA occlusion, thrombus formation, device position, and peri‐device leak (PDL). PDLs were defined as no PDL (0 mm), minor PDL (≤5 mm), and major PDL (>5 mm). In patients with no PDL or minor PDL, they switched to antiplatelet therapy (aspirin 100 mg daily+ clopidogrel 75 mg daily) until 6 months postprocedure, when aspirin (100 mg daily) alone was continued indefinitely. In patients with major PDL or device‐related thrombus (DRT), anticoagulant was continued and TEE/CCT would be repeated every 3 months until thrombus dissolution.

### 2.5 Safety and efficacy outcome

Efficacy endpoints were accessed by procedural success rate, AF recurrence rate, and thromboembolic events (including ischemic stroke, transient ischemic attacks [TIA], and systemic embolism). Safety endpoints included pericardial effusion/cardiac tamponade, DRT, all‐cause death, and major bleeding complications. Procedural success rate was defined as the accomplishment of PVI and successfully sealing of the LAA. AF recurrence was defined as any documented atrial arrhythmia sustained for >30 s after the 3‐month blanking period. Major bleeding complications referred to fatal bleeding, symptomatic bleeding in a critical organ, or bleeding causing a fall in hemoglobin level more than 20 g/L or leading to blood transfusion.^22^

### 2.6 Statistical analysis

Continuous variables were shown as mean ± SD. Categorical variables were given as frequencies and percentages. Characteristic differences between subjects in different age groups at baseline were evaluated using Student's *t*‐test or χ^2^ test accordingly. Mean follow‐up time was estimated using the Kaplan–Meier method. Time to arrhythmia recurrence was estimated using the Kaplan–Meier estimate arrhythmia‐free survival function. The average annual risk of thromboembolic events was calculated as number of events per 100 patient‐years (100‐PY). The expected annual risk of thromboembolic events was calculated based on the CHA2DSVASc score.^23^, ^24^ Differences between groups were tested using the Log‐rank test. All statistical analyses were performed with SPSS Statistics, version 23.0 (IBM Corp.). *p*‐value < 0.05 was considered statistically significant.

## 3 RESULTS

### 3.1 Basic characteristics of the study population

There were 505 patients (mean age 69.5 ± 7.7 years; 230 [45.5%] female) who underwent CA combined with LAAO in a single procedure from 2018 to 2020 in our center. There were 232 (45.9%) paroxysmal AF patients. The mean CHA2DS2VASc score was 3.2 ± 1.4 and HAS‐BLED score was 2.2 ± 1.1. One hundred and ninety‐eight (39.2%) patients received anticoagulation therapy with NOACs or warfarin. The baseline characteristics were described in Table 1. Forty‐six (9.1%) patients were aged ≥80 years old (mean age 82.3 ± 0.3 years), including 21 (45.7%) female patients. Four hundred and fifty‐nine patients were younger than 80 years old (mean age 68.2 ± 6.9 years). Compared with nonoctogenarian group, patients in the octogenarian group were more likely to have paroxysmal AF (25 [54.3%] vs. 207 [45.1%], *p* < 0.001) and higher CHA2DS2VASc score (4.1 ± 1.3 vs. 3.1 ± 1.4, *p* < 0.0001). Other clinical features were similar between the two groups.

**Table 1:** Baseline clinical characteristics of the study population (*N* = 505).

	Total (*n* = 505)	<80 years (*n* = 459)	≥80 years (*n* = 46)	*p‐*Value
Age, mean (SD), years	69.5 (7.7)	68.2 (6.9)	82.3 (0.3)	<0.001
Female, No. (%)	230 (45.5)	209 (45.5)	21 (45.7)	1
BMI, mean (SD), kg/m^2^	25.0 (3.5)	25.0 (3.5)	24.2 (3.2)	0.15
Paroxysmal AF, No. (%)	232 (45.9)	207 (45.1)	25 (54.3)	<0.001
Hypertension, No. (%)	379 (75)	347 (75.6)	32 (69.6)	0.38
Diabetes mellitus, No. (%)	120 (23.8)	109 (23.7)	11 (23.9)	1
Coronary disease, No. (%)	96 (19)	87 (19.0)	9 (19.6)	0.85
Heart failure, No. (%)	44 (8.7)	40 (8.7)	4 (8.7)	1
History of Strok/TIA, No. (%)	134 (26.5)	122 (26.6)	12 (26.1)	1
CHA2DS2VASc score, mean (SD)	3.2 (1.4)	3.1 (1.4)	4.1 (1.3)	<0.001
HAS‐BLED score, mean (SD)	2.2 (1.1)	2.2 (1.1)	2.2 (0.9)	0.98
Antiarrhythmic therapy, No. (%)				
Class I, No. (%)	30 (5.9)	27 (5.9)	3 (6.5)	0.75
Class II, No. (%)	188 (37.2)	173 (37.7)	15 (32.6)	0.53
Class III, No. (%)	74 (14.7)	67 (14.6)	7 (15.2)	0.83
Anticoagulation, No. (%)				0.31
Warfarin	132 (26.1)	124 (27.0)	8 (17.4)	
NOAC	66 (13.1)	58 (12.6)	8 (17.4)	
Antiplatelet therapy, No. (%)	105 (20.8)	96 (20.9)	9 (19.6)	1
EF, mean (SD), %	63.4 (6.2)	63.3 (6.1)	63.6 (6.5)	0.78
LA, mean (SD), %	42.7 (6.1)	42.7 (6.1)	42.4 (5.5)	0.79
LVDD, mean (SD), %	49.2 (4.9)	49.2 (4.7)	49.3 (6.2)	0.86
LVDS, mean (SD), %	32.4 (4.3)	32.4 (4.4)	32.4 (4.1)	0.99

Abbreviations: BMI, body mass index (calculated as weight in kilograms divided by height in meters squared); LA, left atrium; LVDD, left ventricular end diastolic diameter; LVDS, left ventricular end systolic diameter; LVEF, left ventricular ejection fraction; NOAC, nonvitamin K antagonist oral anticoagulants; TIA, transient ischemic attack.

### 3.2 Procedural details

The combined procedure was successfully achieved in all patients. Additional linear ablation was performed in 320 (63.4%) patients of the study population, including 259 in nonoctogenarian group (56.4%) and 25 in octogenarian group (54.3%). Table 2 displayed detailed parameters of the combined procedure. The mean LAA ostium dimension was 22.8 ± 3.5 mm in the nonoctogenarian group and 23.3 ± 3.6 mm in the octogenarian group (*p* = 0.37). There were no differences in LAA type and Watchman 2.5 device size between the nonoctogenarian and octogenarian groups. The mean RF procedure time was 133.0 ± 42.9 min in the nonoctogenarian group and 129.0 ± 41.3 min in the octogenarian group (*p* = 0.55). The mean LAAO procedure time 33.5 ± 18.5 min in the nonoctogenarian group and 35.7 ± 22.6 min in the octogenarian group (*p* = 0.44). Postprocedure, all patients were prescribed an oral anticoagulant (177 (35%) warfarin, 117 (23.2%) dabigatran, and 211 (41.8%) rivaroxaban). Type of anticoagulant was similar between different age groups.

**Table 2:** Procedural details of the study population between different groups.

	Total (*n* = 505)	<80 years (*n* = 459)	≥80 years (*n* = 46)	*p*‐Value
Procedure, No. (%)				0.2
PVI	185 (36.6)	164 (35.7)	21 (45.7)	
PVI + linear ablation	320 (63.4)	259 (64.3)	25 (54.3)	
LAA ostium, mean (SD), mm	22.8 (3.5)	22.8 (3.5)	23.3 (3.6)	0.37
LAA type, No. (%)				0.39
Chicken‐wing	69 (13.7)	65 (14.2)	4 (8.7)	
Wind sock	13 (2.6)	13 (2.8)	0 (0)	
Cauliflower	419 (83)	377 (82.1)	42 (91.3)	
Cactus	4 (0.8)	4 (0.87)	0 (0)	
Device size, No. (%), mm				0.094
21	36 (7.1)	33 (7.2)	3 (6.5)	
24	101 (20)	90 (19.6)	11 (23.9)	
27	156 (31)	150 (32.7)	7 (15.2)	
30	105 (20.8)	90 (19.6)	15 (32.6)	
33	106 (21)	96 (20.9)	10 (21.7)	
Procedure time, mean (SD), min				
CA	132.6 (42.8)	133.0 (42.9)	129.0 (41.3)	0.55
LAAO	33.7 (18.9)	33.5 (18.5)	35.7 (22.6)	0.44
Anticoagulation after procedure, No. (%)				0.94
Warfarin	177 (35.0)	162 (35.3)	15 (32.6)	
Dabigatran	117 (23.2)	106 (23.1)	11 (23.9)	
Rivaroxaban	211 (41.8)	191 (41.6)	20 (43.5)	

Abbreviations: CA, catheter ablation; LAA, left atrial appendage; LAAO, left atrial appendage occlusion; PVI, pulmonary vein isolation.

### 3.3 Peri‐procedural safety and outcomes at follow‐up

There were 6 cases (1.2%) of pericardial effusion in nonoctogenarian group. In four cases, postprocedural echocardiography showed small pericardial effusion and they recovered during follow‐up without specific treatment. The other two patients developed cardiac tamponade and were successfully treated by pericardiocentesis. No pericardial effusion happened in the octogenarian group. TEE or CCT was performed in 423 (83.8%) and 14 (2.8%) patients 3 months after the procedure, respectively. Three hundred and eight (77.6%) patients obtained no PDL in nonoctogenarian group and 32(80%) in octogenarian group. Minor PDL was found in 89 patients (22.4%) in nonoctogenarian group and 8 (20%) in octogenarian group. No patients had major PDL. DRT was found in four nonoctogenarian patients and one octogenarian patient and oral anticoagulant was maintained in these patients. At 6 months follow‐up, TEE was repeated and showed no thrombus on device in these patients and then, they switched to antiplatelet therapy. Table 3 showed the peri‐procedural and follow‐up outcomes.

**Table 3:** Follow‐up outcomes.

	Total (*n* = 505)	<80 years (*n* = 459)	≥80 years (*n* = 46)	*p*‐Value
3‐month follow‐up TEE/CCT, No. (%)	437 (86.5)	397 (86.5)	40 (87)	1
PDL				0.84
No PDL	340 (77.8)	308 (77.6)	32 (80)	
Minor PDL	97 (22.2)	89 (22.4)	8 (20)	
Major PDL	0 (0)	0 (0)	0 (0)	
Device‐related thrombus, No. (%)	5 (1.0)	4 (0.9)	1 (2.1)	0.38
Mean follow‐up time, M	26.9 ± 9.1	27.1 ± 9.1	25.8 ± 8.7	0.7
Pericardial effusion/cardiac tamponade, No. (%)	6 (1.2)	6 (1.3)	0 (0)	1
AF recurrence, No. (%)	113 (22.4)	103 (22.4)	10 (21.7)	0.99
Stroke/TIA, No. (%)	10 (1.2)	8 (1.7)	2 (4.3)	0.23
Major bleeding, No. (%)	1 (0.2)	0 (0)	1 (2.1)	0.091
All‐cause death, No. (%)	16 (3.2)	16 (3.5)	0 (0)	0.38

Abbreviations: AF, atrial fibrillation; CCT, cardiac computed tomography; M, month; PDL, peri‐device leak; TEE, transesophageal echocardiography; TIA, transient ischemic attack.

**Figure 1:**
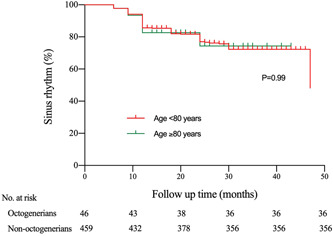
Kaplan–Meier estimates of arrhythmia‐free survival. After a mean follow‐up of 26.9 ± 9.1 months, atrial fibrillation (AF) was documented in 10 (21.7%) patients in octogenarian group and in 103 (22.4%) patients in nonoctogenarian group (*p* = 0.99).

**Figure 2:**
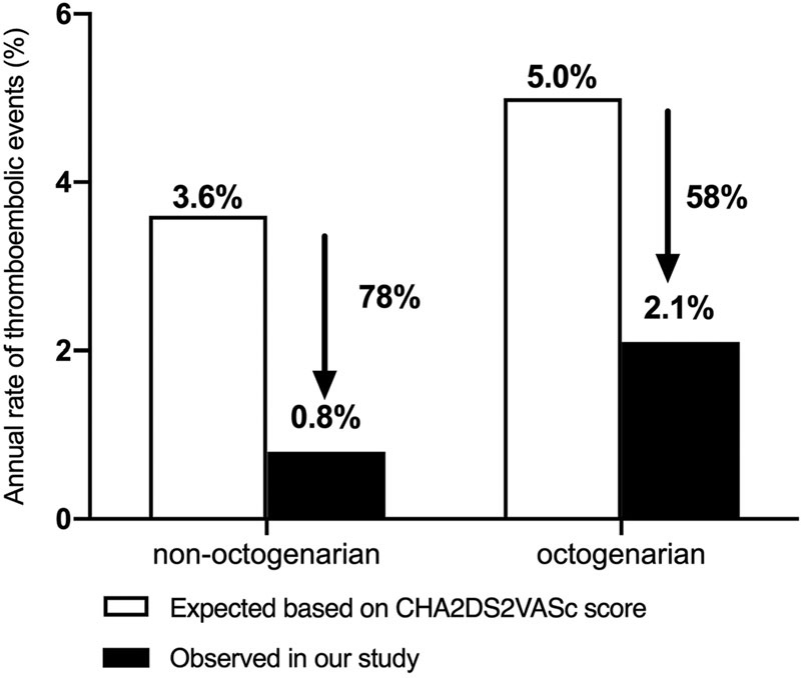
Observed rate of annual thromboembolic events versus the expected rate based on the CHA2DS2VASc score. According to the CHA2DS2VASc score, the estimated thromboembolic risk was 5.0% in the octogenarian group and 3.6% in the nonoctogenarian group. Based on our data, the annual thromboembolic risk was 2.1% and 0.8% in the octogenarian group and nonoctogenarian group, respectively. The annual thromboembolic events decreased 58% in the octogenarian group and 78% in the nonoctogenarian group.

The mean follow‐up time was 26.9 ± 9.1 months (27.1 ± 9.1 months in nonoctogenarian group and 25.8 ± 8.7 months in octogenarian group, *p* = 0.7). The overall AF recurrence rate was 22.4% (113/505), involving 103 (22.4%) patients in nonoctogenarian group and 10 (21.7%) in octogenarian group (*p* = 0.99). The Kaplan–Meier curve of arrhythmia‐free survival was shown in Figure 1. There was no significant difference in term of thrombus events between different age groups (two in octogenarian patients vs. eight in nonoctogenarian patients, *p* = 0.23). In the octogenarian group, one patient underwent TIA 1 day after the procedure when he was on low‐molecular‐weight heparin. Another patient underwent stroke 1 month after the procedure when he was on anticoagulant. In the nonoctogenarian group, TIA occurred in two patients (one happened 1 day after the procedure when he was on low‐molecular‐weight heparin and another happened 5 days after the procedure and ischemic stroke occurred in 6 cases (two happened within 3 months after the procedure while they were on anticoagulant and four of them happened at 1 year after the procedure while they were on antiplatelet therapy. In all these cases, no DRT or leak was evidenced at 3 months TEE follow‐up. None of them were accompanied by long‐lasting stroke‐related physical disabilities. According to the CHA2DS2VASc score, the estimated thromboembolic risk was 5.0% in the octogenarian group and 3.6% in the nonoctogenarian group. Based on our data, the annual thromboembolic risk was 2.1% and 0.8% in the octogenarian group and nonoctogenarian group, respectively. The annual thromboembolic events decreased 58% in the octogenarian group and 78% in the nonoctogenarian group (Figure 2).

No patient died in the octogenarian group, compared with 16 death in the nonoctogenarian group (*p* = 0.38). One major bleeding was recorded in the octogenarian group. The patient was an 80 years old female and suffered stomach bleeding 1 year after the procedure while using aspirin. Her hemoglobin dropped to 42 g/L and gastroscope confirmed there was active bleeding around the gastric ulcer. She received blood transfusion and hemoglobin was 82 g/L at discharge after 1‐week hospitalization.

## 4 DISCUSSION

To the best of our knowledge, this is the first study to assess the efficacy and safety of the CA combined with LAAO in patients aged ≥80 years. Our data suggested that the strategy of ablation combined with LAAO can be performed safely and successfully in octogenarian AF patients as in nonoctogenarian AF patients.

Based on our data, 78.3% octogenarian patients were free from AF and this is comparable to younger patients. Similarly, Swaans et al. first reported that 70% patients were free from AF after 1‐year follow‐up.^10^ A meta‐analysis reported an AF recurrence rate of 24% at an average follow‐up of 21 months.^25^ Most studies regarding combined strategy are based on small sample size and few studies enrolled elderly patients especially those aged ≥80 years. Our analysis included a larger patient cohort and more elderly patients. Histological study revealed that aging is not associated with more fibrosis in left atrium.^26^ In other words, elderly AF patients can benefit from CA equally to younger AF patients. In fact, success rate of single CA procedure in elderly AF patients from our center was similar with the previously reported data, which ranged from 64% to 70%.^17^, ^27^, ^28^

Elderly patients face the highest risk of both stroke and major bleeding. Successful LAAO might thus provide the maximum benefit in this patient population.^25^, ^29^, ^30^ Data from the AMPLATZER Cardiac Plug multicenter registry revealed a similar stroke rates between patients aged <75 years and those ≥75 years.^29^ Yu et al.^25^ analyzed 351 elderly patients implanted with WM device or Amplatzer cardiac plug (ACP) device. They reported an annual thromboembolic events rate of 3.2% in advanced‐age group and 2.1% in younger patients. All patients in our study were implanted with the 2.5 Watchman device. We showed an increasing trend of thrombus events in the octogenarian group and the event risk decreased in both age groups compared with the risk based on the CHA2DS2VASc score. During follow‐up, one DRT was recorded in the octogenarian patients, and this patient did not develop stroke/TIA. Consistently, patients with or without DRT had similar annual rates of stroke/TIA/SE in the EWOLUTION Trial.^7^ Interestingly, all the stroke cases had no leaks during follow‐up. We observed 22.2% cases had minor PDL and no case had major PDL in the combined procedure. This is comparable with previous studies, which reported an incidence of PDL ranging from 5% to 35% in single LAAO procedure.^31^, ^32^ We agreed that PDL size was not associated with an increased risk of thromboembolic events.^33^, ^34^, ^35^ However, data from the NCDR‐LAAO Registry announced patients with PDL < 5 mm had 1.52‐fold higher incidences of thromboembolic and bleeding events.^36^ The pooled analyses of PROTECT AF, PREVEIL and CAP2 studies reported that PDL at 1‐year, but not 45 days, was linked with a twofold increase in ischemic stroke at 5 years.^37^ Due to the short follow‐up time and low event rates, our study is not sufficient to clarify the role of the leaks after the combined procedure.

One major concern for octogenarian patients is that they might face more procedure‐related complications. Based on our experience, the combined procedure did not increase acute or long‐term complications in octogenarian AF patients. In contrast, all pericardial effusion/cardiac tamponade was observed in the nonoctogenarian group. This may be related to the ablation‐related injury and it seems that ablation strategy in the younger group was more aggressive. Moreover, there are a trend of more death in the nonoctogenarian group. In this analysis, the sample size of octogenarian patients is relatively small, and prevalence rates of comorbidities, LVEF and LA values were compared with nonoctogenarian patients. This suggested that the majority of the octogenarians in this cohort were highly selected and biologically relative “healthier” than nonoctogenarian AF patients. Elderly patients with more comorbidities, those who cannot tolerate the procedure or physically weakness might not be enrolled for the procedure. Thus, one should be careful when choosing elderly candidate for CA combined with LAAO.

### 4.1 Limitations

The results of our analyses should be interpreted in the context of several limitations. This is a retrospective analysis performed in a single center with consecutive patients and a relatively short follow‐up. The AF recurrence is mainly accessed by self‐reported symptoms and the Holter monitoring, and its rate may be underestimated. Longer monitoring period such as 14‐days Holter or implantable loops are recommended. The majority of the octogenarians we included were biologically healthy. Also, we did not compare octogenarians undergoing CA with or with not LAAO. Future studies are needed to evaluate the effects of combined strategy even in octogenarians with more challenging clinical conditions.

## 5 CONCLUSION

Our results indicate that CA combined with LAAO in a single procedure seems to be a safe and effective strategy in both nonoctogenarian and octogenarian AF patients. Future study is needed to validate this finding with larger patient cohort.

## CONFLICT OF INTEREST STATEMENT

The authors declare no conflict of interest.

## Data Availability

The data are not publicly available because the containing information could compromise the privacy of research participants. The data will be shared on reasonable request to the corresponding author.
